# Supplementary material for: Relationship between gender roles, motherhood beliefs and mental health
Source: PLoS One. 2024 Mar 20;19(3):e0298750. doi: 10.1371/journal.pone.0298750 (PMC10954095; doi:10.1371/journal.pone.0298750)
Supplement: Attachment — Submitted filename: PLOS ONE Review RR-1-21-24.docx [file pone.0298750.s002.docx]

Review of “Relationship between gender roles, motherhood beliefs, and mental health”

PLOS ONE

The authors have responded thoroughly and graciously to all my previous concerns regarding the manuscript. My remaining comments are minor.

1) It may be helpful for potential readers if the title includes that the study was conducted in Mexico.
2) The term “breeding” could be replaced with an academic term applicable to humans in particular (e.g., “procreation”, “reproduction”, or “bearing children”).
3) The authors have now included information on employment, but I would not describe it as data on “occupation” per se. The data reported Table 3 for Study 3 is more appropriately referred to as “employment status.” On the other hand, occupational data would, for instance, distinguish between professional or “white collar” occupations and “blue collar” occupations.
4) After thoughtful incorporation of reviewers’ feedback, with revisions, the paper is now 70 pages long. The detailed attention to the reviewer feedback is laudable. However, I might suggest moving some tables to an online Appendix section if the paper’s length becomes an issue.

Thank you again for the opportunity to review this important work.
